# Highly Efficient Generation of Transgenically Augmented CAR NK Cells Overexpressing CXCR4

**DOI:** 10.3389/fimmu.2020.02028

**Published:** 2020-08-28

**Authors:** Arezoo Jamali, Jamshid Hadjati, Zahra Madjd, Hamid Reza Mirzaei, Frederic B. Thalheimer, Shiwani Agarwal, Halvard Bonig, Evelyn Ullrich, Jessica Hartmann

**Affiliations:** ^1^Faculty of Advanced Technologies in Medicine, Department of Molecular Medicine, Iran University of Medical Sciences, Tehran, Iran; ^2^Molecular Biotechnology and Gene Therapy, Paul-Ehrlich-Institut, Langen, Germany; ^3^Experimental Immunology, Division of Stem Cell Transplantation and Immunology, Childrens Hospital, Goethe University, Frankfurt, Germany; ^4^Department of Medical Immunology, School of Medicine, Tehran University of Medical Sciences, Tehran, Iran; ^5^Oncopathology Research Center, Iran University of Medical Sciences, Tehran, Iran; ^6^Institute for Transfusion Medicine and Immunohematology, Goethe University, Frankfurt, Germany; ^7^German Red Cross Blood Service Baden-Württemberg-Hessen, Frankfurt, Germany; ^8^Department of Medicine, Division of Hematology, University of Washington School of Medicine, Seattle, WA, United States; ^9^German Cancer Consortium, German Cancer Research Center (DKFZ), Heidelberg, Germany; ^10^Frankfurt Cancer Institute, Frankfurt, Germany; ^11^Division of Molecular Biotechnology, Paul-Ehrlich-Institut, Langen, Germany

**Keywords:** natural killer cell, chimeric antigen receptor, immunotherapy, fully human CAR, CD19, huCAR19, transgenically augmented CAR NK cell, chemokine receptor 4

## Abstract

Natural killer (NK) cells are a noteworthy lymphocyte subset in cancer adoptive cell therapy. NK cells initiate innate immune responses against infections and malignancies with natural cytotoxicity, which is independent of foreign antigen recognition. Based on these substantive features, genetically modifying NK cells is among the prime goals in immunotherapy but is currently difficult to achieve. Recently, we reported a fully human CAR19 construct (huCAR19) with remarkable function in gene-modified T-cells. Here, we show efficient and stable gene delivery of huCAR19 to primary human NK cells using lentiviral vectors with transduction efficiencies comparable to those achieved with NK cell lines. These huCAR19 NK cells display specific and potent cytotoxic activity against target cells. To improve homing of NK cells to the bone marrow, we augmented huCAR19 NK cells with the human CXCR4 gene, resulting in transgenically augmented CAR NK cells (TRACKs). Compared to conventional CAR NK cells, TRACKs exhibit enhanced migration capacity in response to recombinant SDF-1 or bone marrow stromal cells while retaining functional and cytolytic activity against target cells. Based on these promising findings, TRACKs may become a novel candidate for immunotherapeutic strategies in clinical applications.

## Introduction

Cancer immunotherapy using CD19-specific chimeric antigen receptor (CAR) modified T-cells (CAR T-cells) has shown remarkable clinical benefit in patients with relapsed or refractory B-cell malignancies. The first CAR T-cell products, tisagenlecleucel (Kymriah, Novartis) and axicabtagene ciloleucel (Yescarta, Gilead), were approved in the United States in 2017 and Europe in 2018. However, so far, this success has not been translated to other hematological indications, such as acute myeloid leukemia (AML) or even solid tumors. In addition, CAR T-cell therapies can be associated with severe, sometimes even life-threatening adverse events, such as cytokine release syndrome (CRS), neurotoxicity, B-cell aplasia, and/or graft-versus-host disease (GvHD) (1).

Another immune cell type that can recognize and destroy cancer cells is natural killer (NK) cells. Matching their name, this small subset of lymphocytes initiates the innate immune response against infections and malignancies with its natural cytotoxicity behavior in hand. NK cells rely on germline encoded inhibitory and activating receptors sensing a misbalance of the corresponding ligands on the target cell surface and are, therefore, independent of foreign antigen recognition. In recent years, NK cell–based immunotherapy approaches have been developed for the treatment of various malignancies in both autologous and allogeneic settings. The transfer of *ex vivo* expanded autologous and allogeneic NK cells has been found to be safe and well tolerated in a range of clinical trials with no signs of GvHD, CRS, or neurotoxicity, but the effect on tumor suppression appears to be low for autologous NK cell infusions or highly dependent on the type of cancer for allogeneic NK cell infusions (2).

Similar to T-cells, NK cells can be genetically modified with CAR genes to improve their antitumor potential. CAR expression on the surface of NK cells mimics an activating receptor providing a strong activation signal upon contact with its respective ligand on the tumor cell, resulting in CAR NK cell activation and target cell lysis. Considering the beneficial safety profile of NK cells and the possibility to use them as an “off-the-shelf” product in an allogeneic setting make CAR NK cells an attractive alternative to CAR T-cells. However, compared to T-cells, NK cells are harder to modify with viral vectors due to their intrinsic antiviral defense mechanisms (3). Transduction efficiencies of NK cells vary tremendously depending on the cell source, the viral vector system, and the transduction enhancer used (4). Lentiviral vectors (LVs) pseudotyped with the glycoprotein of the vesicular stomatitis virus (VSV-G) are classically used to generate CAR T-cells but are less efficient for NK cells with transduction efficiencies of 20–40% (5, 6). Therefore, optimization of gene transfer protocols for VSV-G pseudotyped LV for the generation of CAR NK cells is urgently required.

Today, fewer than 15 clinical trials using CAR NK cells are performed worldwide, which is far below the amount of ongoing CAR T-cell trials (>200) (1, 7). Similar to CAR T-cell trials, most CAR NK cell trials use a CD19-specific CAR molecule derived from the murine antibody FMC63 (8, 9). Due to the potential of murine-derived targeting domains causing anaphylaxis, more and more CAR T-cell studies are investigating humanized or fully human CAR constructs (1). In this respect, recently, fully human CD19-specific and fully human mesothelin-specific CAR constructs were reported showing efficient expression and strong antitumor activity in T-cells (10–13). Because murine-derived CAR molecules possess the same risk of causing anaphylaxis in engineered CAR NK cells, CAR NK cell approaches should evaluate the feasibility of humanized or fully human CAR constructs as well.

Persistent tumor cells in the bone marrow are a major cause of cancer relapse in several indications including AML (14–16). Trafficking of NK cells to and from the bone marrow essentially depends on chemotaxis. NK cells basically complete their maturation steps in the bone marrow, are retained there, or egress to and emerge in the blood circulation (17). Retention of NK cells in the bone marrow is primarily driven by the interaction of the CXCR4 chemokine receptor expressed on NK cells and its ligands SIP5 (sphingosine-1 phosphate receptor 5) and CXCL12, also known as SDF-1 (stromal cell–derived factor 1), abundantly represented by bone marrow stromal cells. During NK cell maturation, CXCR4 expression decreases, promoting NK cell release from the bone marrow (18). It has been shown that NK cell chemotaxis to the bone marrow is induced by SDF-1 and can be prevented utilizing AMD3100, a CXCR4 antagonist (Plerixafor), resulting in an increase of NK cells in the spleen and other peripheral organs (17, 18). In this respect, it was recently demonstrated that the expression of CXCR4 in NK cells can improve their homing to the bone marrow (19). Combined expression of CAR and CXCR4 might, therefore, enable enhanced chemotaxis of CAR NK cells to the bone marrow allowing more efficient targeting of persistent tumor cells in the tumor niche environment.

To our knowledge, we report here for the first time the highly efficient transduction of primary NK cells with VSV-G pseudotyped LVs delivering a fully human, CD19-specific CAR construct (huCAR19). Generated huCAR19 NK cells are shown to exhibit strong and specific antitumor activity. To augment homing to the bone marrow, NK cells were modified to express human CXCR4 in addition to the huCAR19 construct. These *transgenically augmented CAR NK cells* (TRACKs) are proven to overexpress CXCR4, which leads to enhanced chemotaxis toward SDF-1 and bone marrow stromal cells without any loss in their CD19-specific cytotoxic activity.

## Materials and Methods

### Ethics Statement

This research was approved by the Ethics Committee of the University Hospital Frankfurt, Germany. Written informed consent was obtained from each donor.

### Cell Lines and Primary Cells

HEK-293T (ATCC CRL-11268) and HT1080 (ATCC CCL-121) cells were grown in Dulbecco’s modified Eagle’s medium (DMEM; Biowest, Nuaillé, France) supplemented with 10% fetal calf serum (FCS; Biochrom, Berlin, Germany) and 2 mM L-glutamine (Sigma-Aldrich, Munich, Germany). Nalm-6 [peripheral blood derived B cell precursor leukemia cells (ALL), CD19^+^], JeKo [peripheral blood derived B cell lymphoma cells (non-Hodgkins lymphoma), CD19^+^], CD19-negative JeKo (CD19^*neg*^JeKo), SUP-B15 [bone marrow derived B cell precursor leukemia cells (ALL), CD19^+^], and BV-173 [peripheral blood derived B cell precursor leukemia cells (chronic myeloid leukemia), CD19^+^] cell lines were grown in RPMI 1640 (Biowest, Nuaillé, France) supplemented with 10% FCS and 2 mM L-glutamine. NK92, a malignant non-Hodgkins lymphoma cell line (ATCC^®^ CRL-2407), and NKL, a large granulocyte leukemia cell line (20), were cultivated in X-Vivo 10 medium (Lonza, Belgium) supplemented with 5% human serum (Sigma, United States) and 100 IU/ml of IL-2 (Miltenyi Biotec, Bergisch-Gladbach, Germany). All cell lines were cultivated at 37°C, 5% CO_2_, and 95% humidity for up to 1 month. Cells were split every 2 to 3 days and did not undergo more than 20 passages. Regular testing for Mycoplasma was performed for all cell lines using a PCR Mycoplasma Test Kit (PanReacApplichem, Germany).

Human PBMCs were isolated from the fresh blood of healthy anonymous donors or buffy coats purchased from the German blood donation center (DRK-Blutspendedienst Hessen, Frankfurt, Germany). Primary NK cells were purified by negative selection using the NK cell isolation kit according to the manufacturer’s instructions (Miltenyi Biotec, Bergisch-Gladbach, Germany). After NK cell isolation, 2 × 10^6^ cells were cultivated per well in a 24-well plate in 400 μl of X-Vivo 10 (Lonza, Belgium) medium supplemented with 5% human serum (Sigma, United States) and 100 IU/ml of IL-2 (Miltenyi Biotec, Bergisch-Gladbach, Germany) for at least 72 h at 37°C. The purity of NK cells after isolation was determined by flow cytometry (Supplementary Figures S1B,C).

### Transgene Constructs

The fully human anti-CD19-CAR construct (kindly provided by Dr. Brian G. Till, Fred Hutchinson Cancer Research Center, Seattle, WA, United States) was previously described (11). In brief, the huCAR19 construct consists of the encoding sequences of the VL and VH region of the human 21 Da antibody linked by a 16 amino acid peptides followed by a hinge domain derived from human CD8a, the transmembrane and costimulatory domain of human 4-1BB and the signaling domain of CD3ζ. For the generation of the huCAR19.CXCR4 construct, the human CXCR4 coding sequence was cloned downstream to the huCAR19 sequence linked via self-cleaving peptide P2A. Both constructs reside in the clinical grade lentiviral expression vector SINpWPT (21) and are under the control of a human elongation factor-1 alpha (EF1α) promoter.

### Lentiviral Vector Generation, Concentration, and Titration

Vector particles were generated by transient transfection of HEK-293T cells using polyethylenimine (PEI; Sigma-Aldrich, Munich, Germany) and third-generation packaging plasmids as described before (22–24). In brief, 1 day before transfection, 1.5-2 × 10^7^ cells were seeded into a T175 flask. In total, 35 μg DNA was added to 2.3 ml of DMEM without additives and combined with 2.2 ml DMEM containing 140 μl of 18 mM PEI solution. The transfection solution was mixed and incubated for 20 min at room temperature. The culture medium was exchanged to 10 ml DMEM supplemented with 15% FCS and 2 mM L-glutamine before the transfection solution was added to HEK-293T cells. 4–6 h later, the medium was replaced by DMEM supplemented with 10% FCS and 2 mM L-glutamine. 2 days after transfection, the cell culture supernatant was collected, filtered via a 0.45 μm filter, and released vector particles were concentrated by a 20% sucrose cushion at 4500 × *g* for 24 h. The supernatant was discarded, and pellets were resuspended in 60 μl Dulbecco’s Phosphate Buffered Saline (PBS; Lonza, Cologne, Germany) per T175 flask. A third-generation lentiviral packaging system was used to produce CAR-carrying lentiviral particles. Plasmid ratios for the generation of lentiviral particles were described previously (22) and can be found in the Supplementary Table S1.

All produced vector particles were titrated by transducing HT1080 cells with five serial dilutions of vector particles. The expression of huCAR19 transgene was detected 4 days post-transduction by flow cytometry. Titers were calculated as described before (25). Particle numbers were determined using Nano sight NS300 (Malvern Ltd., United Kingdom).

### NK Cell Transduction

For transduction of NK cell lines, 3 × 10^4^ of NK-92 or NKL cells were seeded into a single well of a 96-well flat-bottom plate in 100 μl X-Vivio10 medium supplemented with 5% human serum and 100 IU/ml IL-2 (complete X-Vivo medium). The transduction enhancer polybrene (Sigma) was added to the cells at a concentration of 8 μg/ml before adding 100 μl complete X-Vivo 10 medium containing 2 μl of concentrated vector stock.

For transduction of primary NK cells, 4 × 10^4^ purified NK cells, if not otherwise specified, were seeded into a single well of a 96-well flat-bottom plate in 100 μl complete X-Vivo 10 medium 72 h post NK cell isolation. For each transduction experiment, 2 μl of concentrated vector stock was used. The transduction enhancer polybrene (Sigma-Aldrich, Germany) was utilized at a concentration of 8 μg/ml and added directly to the cells prior to transduction. Vectofusin-1 (Miltenyi Biotec, Germany) was used according to the manufacturer’s instructions and, as described before (26), at a final concentration of 10 μg/ml. In brief, Vectofusin-1 and vector particles were diluted in X-Vivo10 medium without additives in a total volume of 50 μl before both solutions were mixed, incubated for 5–10 min at room temperature, and finally added to the NK cells. Retronectin (Takara, United States) was precoated in a 96-well flat-bottom plate at a concentration of 20 μg/cm^2^ and incubated overnight at 37°C 1 day before transduction. Afterward, Retronectin solution was removed, the plate was blocked with 2% BSA in PBS for 30 min and washed with PBS before seeding NK cells and adding vector particles. If not otherwise specified, polybrene was used as a transduction enhancer.

To increase transduction efficiency, spinfection was performed at 850 × *g* at 32°C for 90 min. Afterward, NK cells were gently resuspended by pipetting. Fresh complete medium was added every other day after transduction until cell analysis. Transgene expression was determined by flow cytometry 3 days post-transduction. The functional capability of transduced cells, including migration assay, cytotoxic activity, cytokine production, and degranulation, was analyzed 4 days after transduction. An overview of the experimental setup is provided in Supplementary Figure S1A.

### Cytotoxicity Assay

Cytotoxic activity of huCAR19-LV or huCAR19.CXC4-LV transduced NK cells was determined using CD19^+^ Nalm-6 cells or other tumor cell lines. CAR expression was detected 72 h post-transduction by flow cytometry, and cytotoxic activity of NK cells was evaluated on the following day. 5 × 10^4^, 2.5 × 10^4^, or 1 × 10^4^ CAR-positive NK cells or untransduced NK cells were cocultured with 1 × 10^4^ Nalm-6 cells that were previously labeled with CellTrace^TM^ CFSE (Invitrogen, United States) according to the manufacturer’s instructions. To compensate for variations of transduction efficiency, the effector cell population was normalized to an absolute NK cell number by the addition of untransduced NK cells. Nalm-6 cells without effector cells were used as control. Coculture was performed for 4 h at 37°C and 5% CO_2_ in a total volume of 200 μl RPMI medium supplemented with 10% FCS and 2 mM L-glutamine. Afterward, the cell mixture was stained for dead cells using the fixable viability dye eFluor 780 (Thermo Fisher Scientific, United States) according to the manufacturer’s instructions and analyzed by flow cytometry. The percentage of dead target cells was analyzed as the CFSE positive, viability dye positive cell population.

### Degranulation and IFNγ Secretion Assay

Four days after transduction, 2 × 10^4^ CAR NK cells were seeded in to a single well of 96-well V-bottom plates together with 2 × 10^4^ Nalm-6 cells in a total volume of 200 μl RPMI medium supplemented with 10% FCS and 2 mM L-glutamine and incubated at 37°C and 5% CO_2_. Untransduced NK cells were used as a control. The anti-CD107a antibody (Biolegend, United States) was added directly after seeding the cells, and a mixture of protein transport inhibitors containing brefeldin A (BD Biosciences, United States), and monensim (BD Biosciences) were added after 1 h of coculture according to the manufacturer’s instruction, and cells were incubated for 3 additional hours. At the end of the coculture, supernatant and cells were separately collected. To determine CD107a expression, NK cells were stained for CD3, CD14, CD19, and CD56 markers as well as for dead cells using fixable viability dye eFluor 506. NK cell degranulation was determined by analyzing CD107a expression as an NK cell degranulation marker on live, CD3^–^, CD14^–^, CD19^–^, and CD56^+^ cells by flow cytometry. Collected supernatants were stored at −80°C and used to detect secreted IFNγ by a Duoset ELISA kit (R&D systems, United States) according to the manufacturer’s protocol.

### Migration Assay

A migration assay utilizing polycarbonate transwell plates with a 6.5 mm diameter and 5 μm pore size (Corning Costar, United States) was used to assess the migration potential of NK cells. 4 days after transduction, 1 × 10^5^ NK cells were loaded in the upper chamber in a total volume of 200 μl X-Vivo10 medium supplemented with 5% human serum and appropriate cytokines. Untransduced NK cells cultured in parallel were used as control. Next, 100 ng/ml of SDF-1 (CXCL12; SinoBiological, United States) diluted in 200 μl X-Vivo10 medium supplemented with 5% human serum (Sigma, United States) and 100 IU/ml of IL-2 were added in the lower well. After 2 h of incubation at 37°C and 5% CO_2_, cells and medium of the lower chamber were harvested, and the number of migrated cells was quantified by flow cytometry analysis. To inhibit chemotaxis, 25 μg/ml AMD3100 (CXCL12 antagonist; Sigma Aldrich, Germany) was added to NK cells for 2 h at 37°C prior to the experiment onset.

To evaluate the cytotoxic functionality of migrated cells, a coculture assay of harvested NK cells with CD19^+^ Nalm-6 cells was performed. For this purpose, migrated cells were collected, counted, and cocultured with Nalm-6 cells at a 1:1 ratio for 4 h at 37°C and 5% CO_2_, followed by washing and staining for viability and CD19. The cytotoxic capacity of migrated NK cells was determined by the decrease in the percentage of living CD19 positive cells analyzed by flow cytometry.

Furthermore, to simulate the migration of NK cells toward the bone marrow niche, a migration assay with primary culture-expanded bone marrow–derived mesenchymal stromal cells (BMSCs) was performed. BMSCs were generated from pooled bone marrow mononuclear cells and expanded to the end of passage 3 as described (27). After thawing, cells were cultivated for 5 days in basal medium containing 10% platelet lysate. 1 day before starting the migration assay, 0.5 × 10^5^ BMSCs were seeded in the lower chamber of a transwell plate. On the next day, the medium was exchanged to 200 μl X-Vivo10 medium without any supplement. Transduced and untransduced NK cells were labeled with CellTrace^TM^ CFSE (Invitrogen, United States) according to the manufacturer’s instructions, and 1 × 10^5^ CSFE-labeled NK cells with or without AMD3100 treatment were loaded into the upper chambers of the transwell. After 2 h of incubation, microscopic pictures from the lower chamber were acquired (Zeiss AxioVert 200 Inverted Fluorescent Microscope, Germany; power x10 magnification), and the number of migrated cells was quantified by flow cytometry analysis.

### Flow Cytometry

Flow cytometry analysis was performed using the MACSQuant^®^ Analyzer 10 (Miltenyi Biotec, Bergisch-Gladbach, Germany), and data were analyzed by FCS Express 6 (*de novo* Software, Glendale, CA, United States). To determine the purity of NK cell purification on the day of isolation, 72 h after culture, and during the final analysis, cells were stained with fixable viability dye eFluor506 (e.bioscience), anti-CD3 (clone BW264/56), anti-CD14 (clone TÜK4), and anti-CD19 (clone LT19) antibodies all conjugated with VioGreen as well as APC-labeled anti-CD56 antibody (all Miltenyi Biotec, Germany). Viable cells that were CD3^–^, CD14^–^, and CD19 negative cells but positive for CD56 were considered to be NK cells according to the gating strategy in Supplementary Figure S1B. CXCR4 was detected using anti-CD184 (clone 12G5; BD Horizon, United States) conjugated to BV421. CAR expression was determined by cell labeling with biotinylated human recombinant CD19 protein (CD19-Fc, Acro Biosystemms, United States) followed by secondary labeling with PE-conjugated Streptavidin (Miltenyi Biotec, Germany). FITC conjugated anti-CD107a (LAMP-1) antibody (Biolegend, United States) was used to evaluate degranulation on NK cells after coculture with target cells. After staining, cells were washed twice with washing buffer (PBS, 2% FCS, 0.1% NaN_3_) and fixed with PBS containing 1% formaldehyde prior to analysis.

### Statistical Analysis

Statistical analyses were performed with Prism 7 software (GraphPad, San Diego, CA, United States). Statistical significance was determined by applying the unpaired two-tailed Student’s *t* test as indicated; *p* values less than 0.05 were considered significant.

## Results

### Efficient huCAR19 Gene Delivery to Primary NK Cells

**FIGURE 1:**
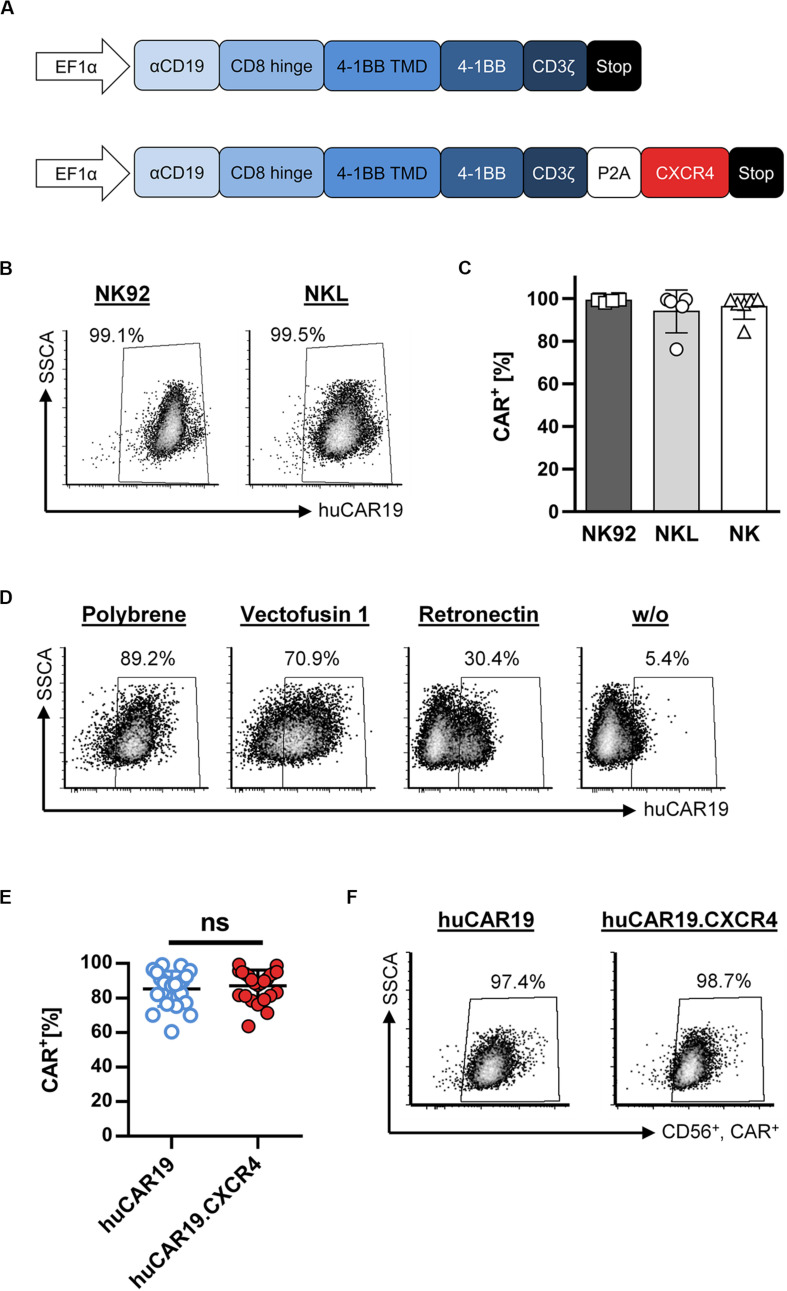
Fully human CAR gene delivery to NK cells. **(A)** Schematic illustration of huCAR19 (top) and huCAR19.CXCR4 (bottom) constructs used to generate CAR NK cells. The second-generation fully human CAR consists of the anti-CD19 scFv derived from the human monoclonal antibody 21 Da (αCD19) linked to the hinge domain of human CD8a (CD8 Hinge), the transmembrane and costimulatory domains of human 4-1BB (4-1BB TMD and 4-1BB), and the signaling domain of CD3ζ. The coding sequence of human CXCR4 is connected to the CAR gene by a P2A self-cleaving peptide. Transgene expression is under control of the human elongation factor 1-α promoter (EFI-α). **(B,C)** NK-92 and NKL cells as well as primary NK cells were transduced with huCAR19-LV particles in the presence of polybrene and analyzed for CAR expression 3 days post-transduction by flow cytometry. The percentage of CD19-CAR-positive cells is indicated. Each transduction experiment was performed at least five times. Representative dot plots for NK-92 and NKL-transduced cells are shown in B. Individual results of transduced NK-92, NKL, and primary NK cells are shown in C as a bar diagram with mean value and SD. **(D)** Effect of different transduction enhancers on huCAR19 gene delivery to primary NK cells; 3 × 10^4^ primary NK cells were transduced with huCAR19-LV particles in the presence or absence of polybrene, Vectofusin-1, and Retronectin. 3 days post-transduction, huCAR19 transgene expression was detected by flow cytometry. Representative dot plots are shown (*n* = 2, with technical replicates). The percentage of huCAR19-positive NK cells is indicated. **(E,F)** Addition of human CXCR4 sequence to the huCAR19 transgene did not impact huCAR19 expression. Primary NK cells of six different donors were transduced with huCAR19-LV or huCAR19.CXCR4-LV particles in the presence of polybrene. The percentage of CD19-CAR-positive cells was analyzed by flow cytometry 3 days post-transduction. Each transduction experiment was performed at least 10 times. Three to four individually produced vector stocks were applied. Individual results as well as mean value, SD, and significance are shown in E. Representative dot plots are shown in F. ns, not significant by unpaired *t* test (*p* = 0.5245).

A fully human, second-generation CD19-specific CAR construct (huCAR19) as well as the huCAR19 construct linked to the human CXCR4 sequence via a P2A site (huCAR19.CXCR4) were used to produce CAR NK cells by lentiviral transduction. The huCAR19 gene cassette contains the scFv regions derived from the human 21D antibody as a binding domain followed by a CD8a hinge domain, the transmembrane, and the costimulatory domain from 4-1BB as well as the intracellular signaling domain of CD3ζ (Figure 1A). LVs encoding both CAR transgenes were produced in several batches. Characteristics of produced LV batches, including titer, particle count, and average size as well as corresponding multiplicity of infection (MOI), are listed in Supplementary Table S2.

First, CAR gene delivery to the NK cell lines NKL and NK-92 was analyzed by applying polybrene as a transduction enhancer, revealing very efficient gene transfer (Figures 1B,C). 3 days after transduction, nearly 100% of the NKL and NK-92 cells were transduced by huCAR19-LV. Next, generated huCAR19-LV particles were applied to transduce purified, primary NK cells in parallel to NKL and NK-92 cells in the presence of polybrene. Surprisingly, the transduction of primary NK cells was as efficient as those of NK cell lines determined by CD19-CAR detection 3 days after vector treatment (Figure 1C). On average 94.4% of NKL, 99.1% of NK-92, and 96.2% of primary NK cells were transduced. An overview of the experimental timeline and gating strategy for primary NK cells is provided in Supplementary Figure S1.

To investigate the effect of different transduction enhancers on gene delivery to primary NK cells, transduction of NK cells was performed in the presence or absence of polybrene, Vectofusin-1, and Retronectin. The most pronounced gene transfer was achieved in the presence of polybrene (nearly 90%) compared to Vectofusin-1 with 70.1% and Retronectin with 30.4% (Figure 1D). Samples without any enhancer showed a moderate transduction rate (5.4%). Therefore, all further experiments were performed using polybrene as an enhancer.

In the next step, we investigated whether adding the human CXCR4 gene sequence to the huCAR19 construct has any adverse effect on huCAR19 expression. Nearly equivalent transduction rates were achieved for both CAR constructs with a mean transduction efficiency of 87.11% for huCAR19-LV and 85.25% for huCAR19.CXCR4-LV (Figures 1E,F). Prolonged cultivation up to 2 weeks post transduction revealed that the CAR constructs were stably expressed on primary NK cells as determined by flow cytometry (Supplementary Figure S2A). These results demonstrate the feasibility of highly efficient CAR gene transfer to primary NK cells by applying LV vectors.

### CXCR4 Expression Can Be Increased on Primary NK Cells Using huCAR19.CXCR4-LV

**FIGURE 2:**
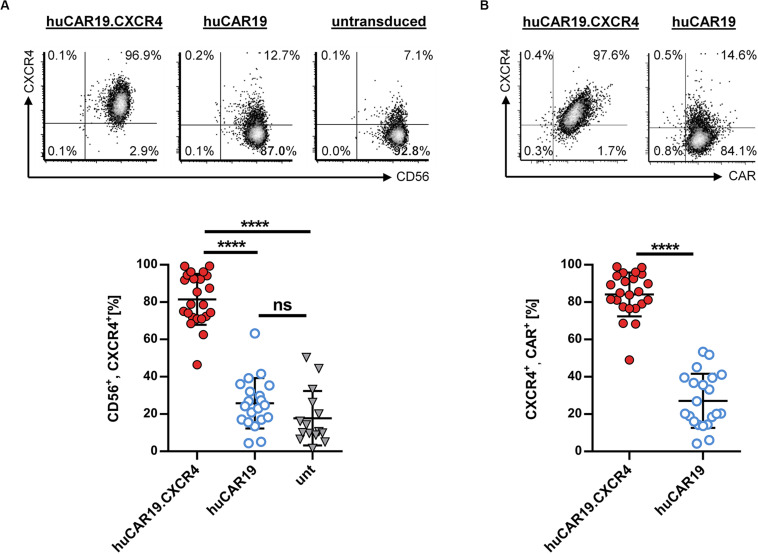
CXCR4 overexpression on NK cells. Primary NK cells were transduced with huCAR19.CXCR4-LV or huCAR19-LV particles and analyzed for CXCR4 expression by flow cytometry 3 days post-transduction. Untransduced (unt) cells were cultured and analyzed in parallel. Each transduction experiment was performed at least 10 times with technical replicates. Three to four individually produced vector stocks were used. Mean value, SD, and significance are indicated. **(A)** Surface expression of CXCR4 on CD56-positive NK cells. Representative dot plots (upper panel) indicate the percentage of cells in each quadrant. Individual results of each experiment for CD56, CXCR4-double positive cells are shown in the lower panel. **(B)** Surface expression of CXCR4 on CD19-CAR-positive NK cells. Representative dot plots (upper panel) indicate the percentage of cells in each quadrant. Individual results of each experiment for CXCR4, CAR-double positive cells are shown in the lower panel. ****p < 0.0001; ns, not significant by unpaired *t* test. Characteristics of isolated, primary NK cells prior to transduction are shown in Supplementary Figure S1.

To enhance the migration capability of CAR NK cells toward tumor cells residing in the bone marrow, we generated huCAR19.CXCR4-LV particles delivering CXCR4 in addition to the CAR construct. To evaluate the ability of the vector to induce CXCR4 overexpression, primary NK cells were isolated from healthy donors and cultivated with IL-2 for 2 days before transduction with huCAR19.CXCR4-LV or huCAR19-LV particles. Prior to transduction, highly purified primary NK cells were less than 17% CXCR4 positive (Supplementary Figure S1C), and 72 h post-transduction, significant CXCR4 expression was detected on NK cells treated with LV particles harboring the huCAR19.CXCR4 construct (on average, 81.5% in the CD56^+^ cell population), and co-expression of both inserts was observed (Figure 2). On average, more than 84% of the cells co-expressed CAR and CXCR4 upon huCAR19.CXCR4 gene delivery (Figure 2B). In contrast, CXCR4 was only expressed on 25.7% of CD56^+^ or 27.1% of huCAR19^+^ NK cells treated with huCAR19-LV particles (Figure 2). Notably, the level of CXCR4 on NK cells transduced with the huCAR19 construct alone was not significantly enhanced and was similar to the levels of untransduced NK cells cultivated in parallel (Figure 2A). Expression of CXCR4 was stable for at least 2 weeks post-transduction as determined by flow cytometry (Supplementary Figures S2B,C). Notably, cell viability, proliferation capacity, and phenotype of the generated CAR NK cells were comparable to untransduced primary NK cells cultured in parallel, demonstrating that the transduction process or transgene expression did not negatively affect NK cell cultivation (Supplementary Figure S3).

### Functional Evaluation of huCAR19 Modified NK Cells

**FIGURE 3:**
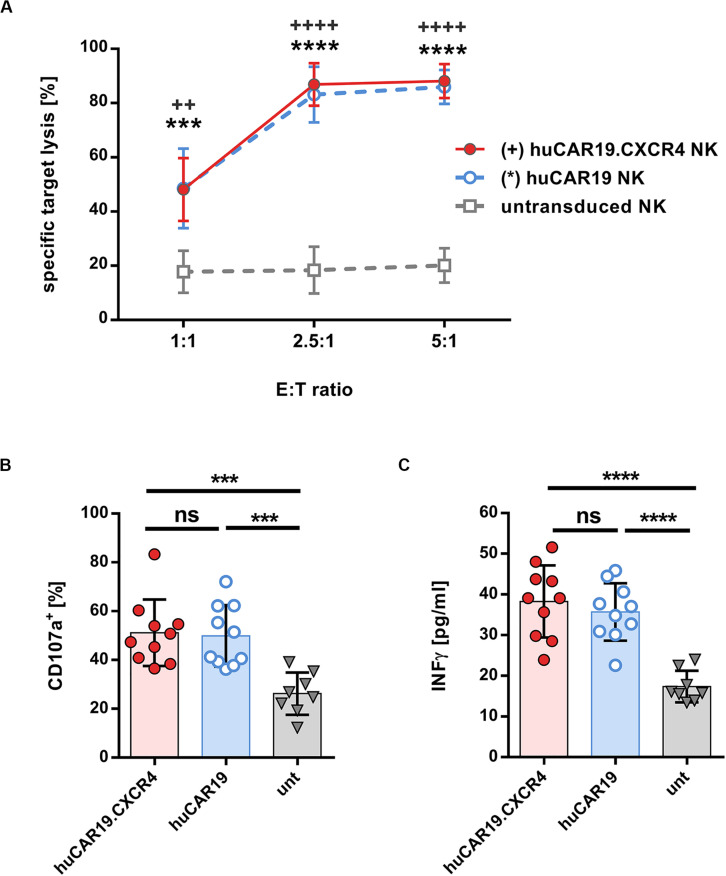
Functional characteristics of gene-modified CAR NK cells. Analysis of cytotoxic function, degranulation, and IFN**γ** secretion of huCAR19 or huCAR19.CXCR4 gene-modified primary NK cells upon cocultivation with CD19-positive Nalm-6 cells. Untransduced primary NK cells cultured in parallel were used as a control. CAR NK cells were generated by transduction of primary NK cells with huCAR19-LV or huCAR19.CXCR4-LV particles at an MOI of 1–5. **(A)** Flow cytometry-based killing assay. After determination of huCAR19 expression, CAR NK cells as well as untransduced primary NK cells were mixed with CFSE-labeled Nalm-6 cells in different effector-to-target (E:T) ratios as indicated. The percentage of dead target cells were identified as double positive cells for CFSE and viability dye by flow cytometry. Mean values, SDs, and significance are shown. *n* = 7 with technical replicates. Unpaired *t* test was performed to determine significance comparing CAR NK cells (^∗^for huCAR19.NK cells; ^+^for huCAR19.CXCR4 NK cells) versus untransduced samples. ^++^*p* < 0.005; ^∗∗∗^*p* < 0.001; and *^∗∗∗^, ^++++^*p* < 0.0001. **(B,C)** Degranulation **(B)** and IFNγ secretion assay **(C)**. Transduced as well as untransduced primary NK cells were mixed with Nalm-6 cells at an E:T ratio of 1. In the first hour of coculture FITC conjugated anti-CD107a antibody was added. After 4 h, cells and supernatant were collected. Harvested cells were used to determine CD107a expression by flow cytometry **(B)**. IFNγ secretion was determined in the supernatant by ELISA **(C)**. Bar diagrams show individual results as well as mean values, SDs, and significance. *n* = 5 with technical replicates. ^∗∗∗^*p* < 0.001; ^****^p < 0.0001; ns, non-significant by unpaired *t* test.

In the next step, the specific cytolytic activity of huCAR19 NK cells augmented with or without CXCR4 was tested. For this purpose, CFSE-labeled CD19^+^ Nalm-6 cells were cocultured with the genetically modified NK cells at a different effector to target ratios for 4 h. In all experiments, at least 63% of the NK cells were CAR positive (Supplementary Figure S4A). Both types of huCAR19 NK cells efficiently killed the tumor cells in all applied ratios (Figure 3A). Already at an effector-to-target ratio of 2.5 to 1, nearly 80% of all tumor cells were lysed by the genetically modified NK cells. In contrast, untransduced NK cells showed only moderate cytotoxic activity against the CD19^+^ Nalm-6 cells (less than 20% dead cells for all tested effector-to-target ratios; Figure 3A). To further demonstrate the specificity of the huCAR19 NK cells, CD19 positive (SUP-B15, BV-173, and JeKo) and negative (CD19^*neg*^JeKo) tumor cell lines were evaluated in a killing assay. SUP-B15, BV-173, and JeKo cells were efficiently lysed by both types of the huCAR19 NK cells, and only background killing was present for CD19^*neg*^JeKo cells or untransduced NK cells (Supplementary Figures S4B–E).

Subsequently, we investigated the activation-induced degranulation and interferon gamma (IFNγ) secretion by the TRACKs upon coincubation with CD19^+^ Nalm-6 cells at an effector-to-target ratio of 1:1. Degranulation was measured by surface detection of the degranulation marker CD107a by flow cytometry and IFNγ secretion by ELISA of the coculture supernatants. The average percentage of degranulation marker after specific target stimulation was comparable for huCAR19 and huCAR19.CXCR4 NK cells (on average, 51.5 and 49.9%, respectively) and significantly higher than CD107a level in untransduced control cells (26.4%; Figure 3B and Supplementary Figure S5). Similarly, IFNγ secreted by huCAR19 and huCAR19.CXCR4 NK cells was significantly higher upon coculture with tumor cells compared to unmodified NK cells although no difference in IFNγ secretion was detected between both CAR NK cell products (Figure 3C). Altogether, these data show that both CAR NK cell products are functional with regard to CAR-dependent specific tumor lysis.

### huCAR19.CXCR4 NK Cells Display Enhanced Chemotaxis Activity

**FIGURE 4:**
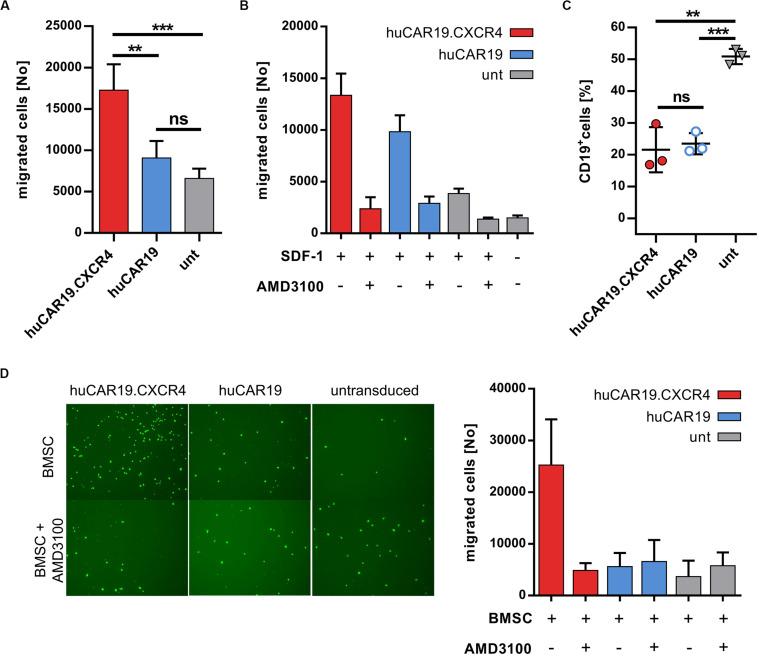
Chemotaxis of NK cells is enhanced by CXCR4 overexpression. The migratory potential of genetically modified NK cells was evaluated in a migration assay using transwell plates. CAR NK cells were generated by transduction of primary NK cells with huCAR19-LV or huCAR19.CXCR4-LV particles. Untransduced primary NK cells cultured in parallel were used as a control. **(A)** Genetically modified or untransduced NK cells were placed in the upper chamber of the transwell plate and analyzed for their potential to migrate toward a lower chamber containing SDF-1 (CXCL12). The number of migrated NK cells was counted by flow cytometry. *n* = 4 with technical replicates. **(B)** Migration potential of NK cells was blocked by the CXCR4 antagonist AMD3100. Untreated or AMD3100 preincubated NK cells were placed in the upper chamber of the transwell plate and analyzed for their potential to migrate in the presence or absence of SDF-1. The number of migrated NK cells was counted by flow cytometry. The experiment was done in technical replicates. **(C)** A coculture assay was performed (in a 1:1 ratio) with migrated NK cells post chemotaxis to determine their cytotoxic potential. Specific target cell lysis was determined by decrease in the percentage of living CD19-positive cells analyzed by flow cytometry. *n* = 3. The experiment was done in technical replicates. Bar diagram show mean value, SD, and significance. **p < 0.005; ***p < 0.001; ns, non-significant by unpaired *t* test. **(D)** The migration potential of CSFE-labeled NK cells with or without AMD3100 pretreatment toward BMSCs plated in the lower wells. Representative fluorescent microscopic pictures of migrated, CFSE-labeled NK cells (right) as well as quantitative data by flow cytometry (left) is shown. Bar diagram shows individual results as well as mean values and standard deviation. The experiment was carried out once with purified NK cells from two different donors.

Next, the ability of CXCR4 gene delivery to enhance NK cell chemotaxis toward CXCL12 was addressed. Migration of huCAR19.CXCR4 NK cells as well as of huCAR19 or untransduced NK cells was determined in a transwell migration assay. Chemotaxis of NK cells from the upper well toward the chemotactic factor SDF-1 in the lower chamber was investigated after 2 h by counting the amount of migrated NK cells. For huCAR19.CXCR4 gene-modified NK cells, the number of migrated cells was nearly twofold enhanced compared to the huCAR19 NK cells and even threefold compared to the untransduced cells (Figure 4A). Untransduced NK cells exhibited a slight tendency to migrate through the transwell even in the absence of SDF-1 (Figure 4B).

To prove that the enhanced migration ability of huCAR19.CXCR4 NK cells is triggered by CXCR4 overexpression, chemotaxis toward SDF-1 was blocked by treating the NK cells with the antagonist AMD3100 for 2 h prior to the migration assay. The number of migrated huCAR19.CXCR4 NK cells after treatment with AMD3100 decreased dramatically from ∼13,100 to ∼2300 cells (roughly a sixfold decline), which is close to the baseline level defined by the amount of untransduced NK cells migrated in the absence of SDF-1 (∼1500; Figure 4B). Notably, the moderate migration ability of huCAR19-LV transduced or untransduced cells was reduced near to baseline levels in the presence of AMD3100 as well (Figure 4B).

To investigate the target-specific lysis potency of transduced cells after migration, a killing assay was performed following an independent transwell migration assay using SDF-1 as the chemotactic factor. In comparison to untransduced cells, significantly higher target cell lysis was observed in coculture with CD19^+^ Nalm-6 cells in a 1:1 ratio by both types of huCAR19 NK cells (Figure 4C).

To further mimic the bone marrow environment and NK cell homing, another migration assay was performed, seeding BMSCs in the lower wells of the transwell plate instead of SDF-1. To discriminate migrated NK cells from BMSCs, NK cells were labeled with CFSE before loading into the upper chamber of the transwell. Fluorescent microscopic imaging and quantification by flow cytometry of migrated cells revealed that the presence of BMSCs is sufficient to induce enhanced chemotaxis of CXCR4-augmented NK cells (more than fourfold compared to huCAR19 NK cells and untransduced cells), which can be abrogated by AMD3100 (Figure 4D and Supplementary Figure S6A). Notably, the addition of recombinant SDF-1 to BMSCs in the lower wells increased the total number of migrated huCAR19.CXCR4 NK cells for at least one donor, but also elevated the levels of migrated huCAR19 NK and untransduced NK cells, which were all close to the background upon AMD3100 treatment (Supplementary Figures S6B,C).

Taken together, these observations demonstrate that huCAR19 NK cells augmented with CXCR4 are able to eradicate CD19^+^ tumor cells in a CAR-dependent manner, have significantly enhanced migration ability, and retain their functional activity post-migration.

## Discussion

Translation of CAR NK cell therapy into clinics is ongoing at a slow pace behind adoptive CAR T-cell therapy owing to some drawbacks regarding not only purification and expansion, but also efficient delivery of transgenes into NK cells and migration capacity (28, 29).

Natural killer cell lines can overcome these limitations, but they have other obstacles with regard to clinical applicability. Although, for basic research, several NK cell lines exist, the only clinically applicable cell line is NK-92. CAR-expressing NK-92 cells are currently under clinical evaluation, but clinical efficacy has to be awaited (30, 31). One major disadvantage of CAR NK-92 cells is their lymphoma origin, requiring irradiation before infusion, limiting their persistence, proliferation, and cytotoxic potential (7). This presents a challenge, especially when attempting to treat rapidly progressing cancers, such as AML. Therefore, CAR NK cells of primary origin are favored over CAR NK-92 cells for certain indications. In the current study, we demonstrate for the first time a highly efficient generation of CAR NK cells derived from peripheral blood encoding fully human CD19-specific CAR augmented by human CXCR4 with potent antitumor activity and migration capability. Genetic modification of primary NK cells was achieved by transduction with third-generation LV particles pseudotyped with VSV-G using polybrene as an enhancer. Interestingly, the transduction of primary NK cells was as efficient as that of the NK cell lines. This quite surprising CAR gene delivery to primary NK cells was not only reproducible in repeated experiments, but it was also stable for at least 2 weeks post-transduction. In addition, cell viability, proliferation capacity, and phenotype were not affected by polybrene or the transgene. This is especially important for the generation of a sufficient number of CAR NK cells for immunotherapy. Further, a high NK cell purity was achieved upon isolation, which is important to avoid T-cell mediated GvHD upon infusion of allogeneic NK cell products (2).

The choice of transduction enhancer and viral envelope protein can tremendously influence transduction efficiencies (26). Direct comparison of Retronectin, Vectofusin-1, and polybrene revealed that Vectofusin-1 and polybrene are beneficial for the transduction of primary NK cells using VSV-G peusdotyped LV particles and clearly outperform Retronectin. Polybrene is also a well-known transduction enhancer in the field of NK cells. High transduction efficiencies can be reached for NK cell lines, but for primary NK cells, gene transfer rates of only 8–55% were reported depending on the viral vector and cell source used (32–34). In our study, on average, more than 80% of gene modified NK cells could be reached. Notably, in contrast to Boissel and colleagues (33), we did not observe a negative effect of polybrene on NK cell viability or proliferation. Similar high gene transfer rates to primary NK cells were reached using Vecotfusin-1 as an enhancer, which might even be increased when using a different viral envelope. For Vectofusin-1, it was recently demonstrated that transgene delivery to primary NK cells can be improved by pseudotyping LVs with the envelope protein from endogenous feline virus (RD114) or baboon endogenous retrovirus (BaEV) instead of VSV-G (5, 6). In the field of CAR T-cells gamma-retroviral vectors or VSV-G pseudotyped LVs are most commonly used for their generation. Therefore, clinical translation of CAR NK cells will benefit from the use of an already approved viral vector, highlighting the value of the demonstrated high transduction efficiency of primary NK cells in the current study with VSV-G pseudotyped LVs (33).

A clinical grade lentiviral expression backbone harboring a fully human CD19-specific CAR construct with or without additional human CXCR4 gene was used to generate huCAR19 and huCAR19.CXCR4 NK cells, respectively. Both CAR NK cells showed potent and specific antitumor activity on CD19-positive patient-derived primary tumor cell lines of various origin, including peripheral blood– or bone marrow–derived B-cell precursor leukemia cells from ALL or chronic myeloid leukemia patients and peripheral blood–derived B-cell lymphoma cells from a patient with non-Hodgkins lymphoma. In addition, upon antigen stimulation, robust INF-γ secretion and upregulation of the degranulation marker CD107a were induced. These results demonstrate that the fully human CD19 CAR construct not only shows strong antitumor activity in T-cells (11), but is also very effective upon introduction to NK cells in terms of cytokine secretion and tumor cell lysis. Notably, the types of proinflammatory cytokines secreted by NK cells differ from those produced by T cells. *Ex vivo* expanded primary human NK cells are known to mainly secrete IFN-γ and GM-CSF upon activation, which are relatively safer than the proinflammatory cytokines produced by T-cells, including TNF-α and IL-6 (35, 36). Secretion of IL-6, IL-1, and TNF-α by CAR T-cells is associated with the development of CRS, a common and severe side effect of CAR T cell therapy. In contrast to CAR T-cells, activated CD19-specific primary CAR NK cells, CAR NK92 cells, or CAR CIK cells were shown by us and others to secrete high levels of IFN-γ and/or GM-CSF but only moderate levels of TNF-α, and production of IL-6 was not observed (37–39). A recent report of a clinical trial did reveal that, among 11 patients with relapsed or refractory CD19-positive malignancies, the majority had a clinical response to treatment with primary CD19-specific CAR NK cells without the development of major toxic effects, including CRS (40), supporting the hypothesis that CAR NK cells might be a safer alternative to CAR T-cells.

Concerning the limited migratory potency of NK cells to malignant cells residing in bone marrow niches or other tumor sites, promoting NK cell chemotaxis via chemokine receptor upregulation has been considered before. Primary NK cells engineered to express the chemokine receptor CCR7 or CXCR2 were demonstrated to have an improved migratory ability toward their respective ligands (41, 42). In an NK cell line, even the combination of CAR and CXCR4 were evaluated, showing that YT2 cells expressing EGFRvIII-CAR and CXCR4 migrate to and specifically kill SDF-1-secreting glioblastoma cells (43). For T-cells, CXCR4 overexpression enhanced their bone marrow migration resulting in local engraftment (44). For NK cells, the power of CXCR4 expression was recently demonstrated by exploring the CXCR4 gain of function mutation present in WHIM (warts, hypogammaglobulinemia, infections, and myelokathexis) syndrome, resulting in an increased NK cell migration toward bone marrow (19, 45). In our study, we could show efficient overexpression of native human CXCR4 on primary CAR NK cells upon genetic engineering. These TRACKs displayed not only strong and specific antitumor activity, but also enhanced chemotaxis toward recombinant SDF-1 and BMSCs. This boosted migratory potential might facilitate an improved clearing of CD19 positive tumor cells residing in the bone marrow *in vivo*, which has to be explored in future work. In conclusion, this work demonstrates that TRACKs could be an important player for NK cell–based adoptive cell therapy and should be further investigated for their translation to clinics.

## Data Availability Statement

All datasets generated for this study are included in the article/Supplementary Material.

## Ethics Statement

The studies involving human participants were reviewed and approved by University Hospital Frankfurt. The patients/participants provided their written informed consent to participate in this study.

## Author Contributions

AJ designed and performed the experiments. AJ and JeH evaluated the data. FT contributed to the experimental design and data evaluation. SA helped with data evaluation. HM contributed to the construct design. EU, HB, FT, and SA contributed protocols and reagents. ZM, JaH, and JeH conceived and designed the study. JeH and EU supervised the work. AJ and JHar prepared the figures and wrote the manuscript. All authors contributed to the article and approved the submitted version.

## Conflict of Interest

The authors declare that the research was conducted in the absence of any commercial or financial relationships that could be construed as a potential conflict of interest.
