# Supplementary material for: Highly Efficient Generation of Transgenically Augmented CAR NK Cells Overexpressing CXCR4
Source: Front Immunol. 2020 Aug 28;11:2028. doi: 10.3389/fimmu.2020.02028 (PMC7483584; doi:10.3389/fimmu.2020.02028)
Supplement: Supplementary file 1 [file Data_Sheet_1.PDF]

Supplementary Material

Supplementary Figures

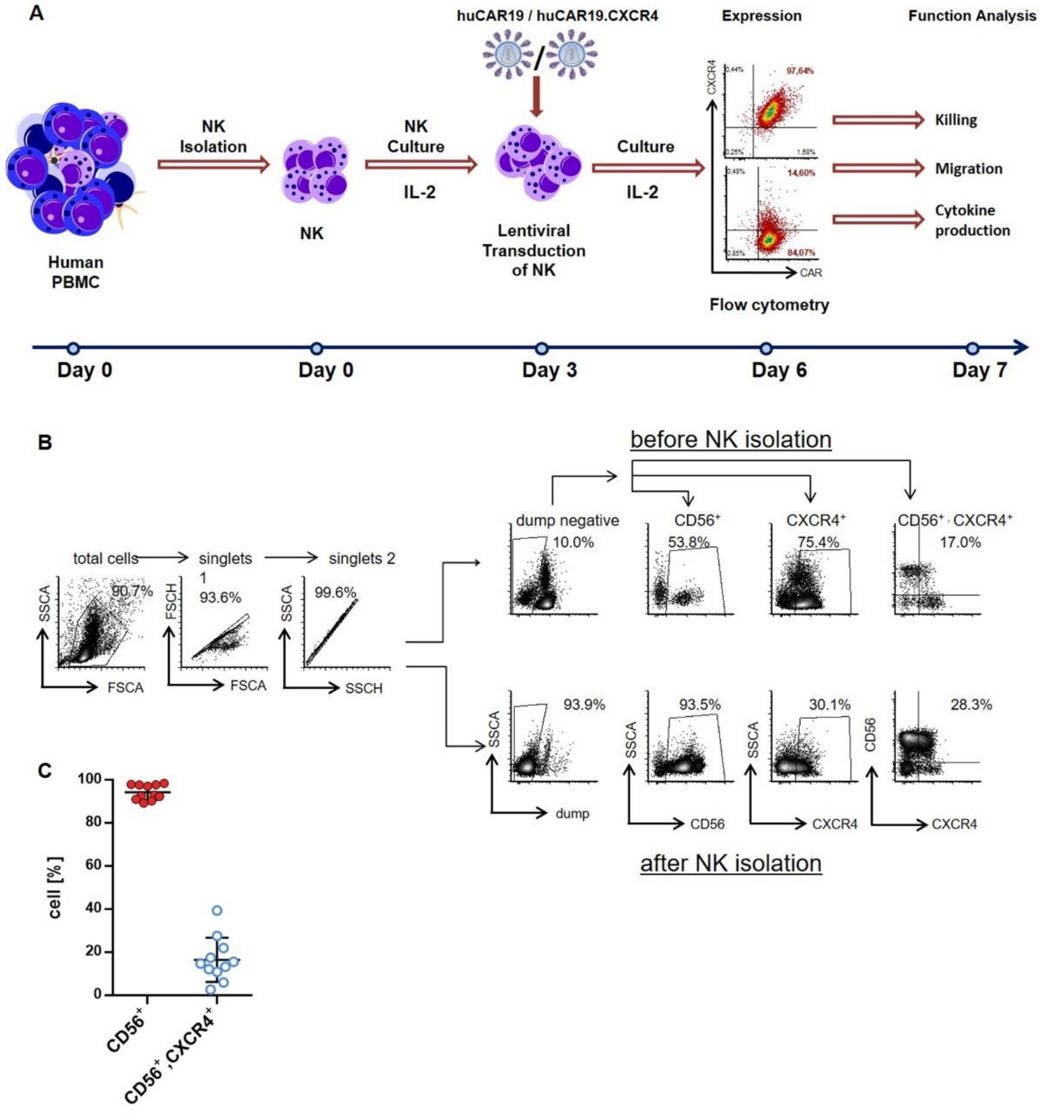

Supplementary Figure S1: The schematic workflow and gating strategy.

(A) Scheme of transduction and analysis procedure including workflow of experimental steps according to the time. NK cells were isolated from human PBMCs by NK cell enrichment and cultivated for 3 days with IL-2 prior to transduction. After transduction, NK cells were cultivated for 3-4 days with IL-2 until functional analysis. **(B)** Gating strategy for primary NK cells prior and after isolation. A dump channel was designed to exclude dead, $CD3^+$, $CD14^+$ and $CD19^+$ cells. Dump channel negative cells were considered as parent gate for viable, single positive $CD56^+$ or $CXCR4^+$ as well as for double positive $CD56^+$, $CXCR4^+$ cells. **(C)** Purity of isolated $CD56^+$ NK cells and percentage of $CD56^+$, $CXCR4^+$ cell population measured by flow cytometry. Data represents results of 11 independent experiments, showing individual values with mean and standard deviations.

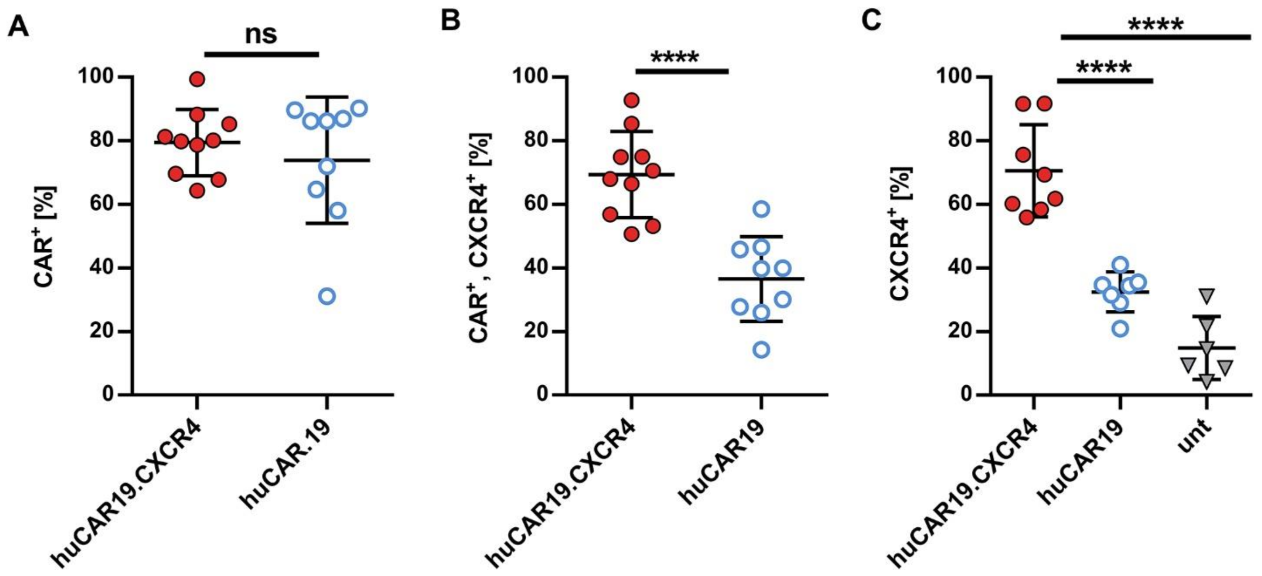

Supplementary Figure S2: Long-term expression of gene of interest by genome engineered primary NK cells.

10-12 days post transduction of NK cells with 2 µl of lentiviral vectors containing either huCAR19 or huCAR19 transgenes the amount of huCAR19 (A), the percent of double positive cells for huCAR19 and CXCR4 expression (B) and the level of CXCR4 in $CD56^+$ cells (C) were detected by flow cytometry. Cultivation of untransduced NK cells (unt) was prolonged up to 10-14 days as well. Dot plots are cumulative data from at least 7 independent experiments. Individual values, with mean, standard deviation and significance are shown. ****$p < 0.0001$; ns, non-significant by unpaired t-test.

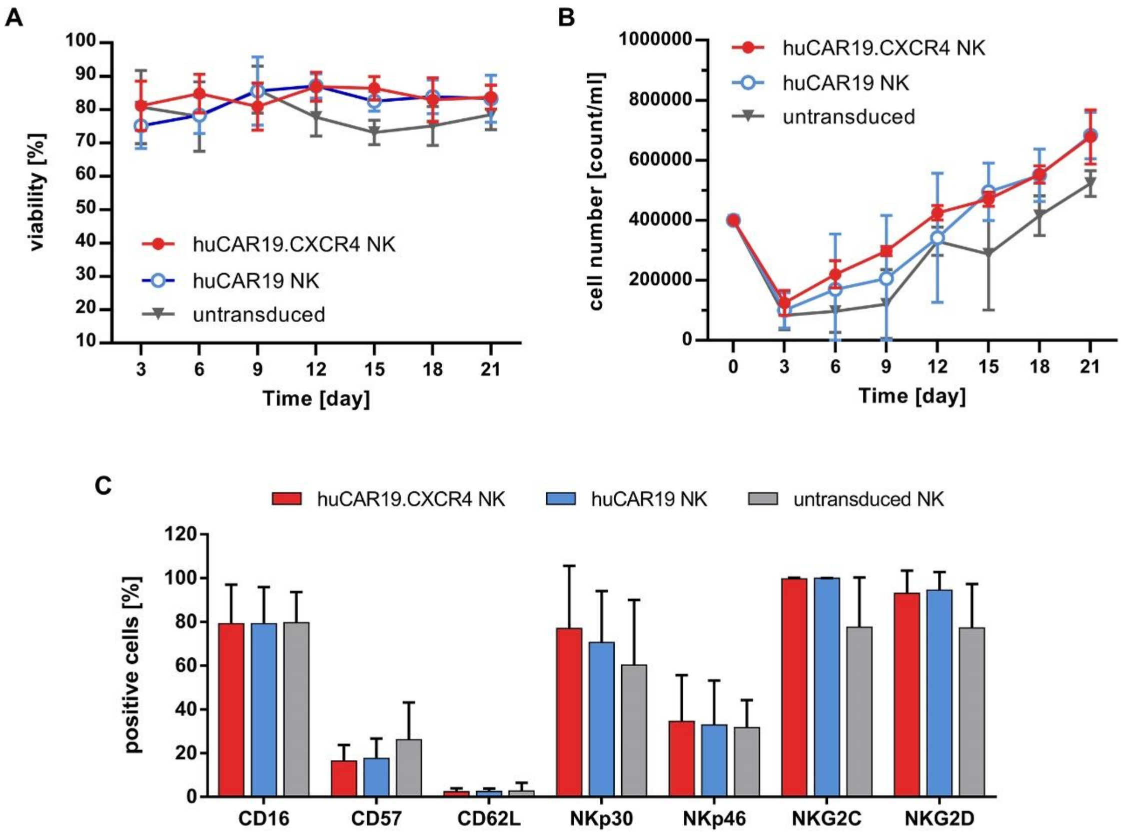

Supplementary Figure S3: Cell viability, proliferation capacity and phenotype of generated CAR NK cells.

After transduction (day 0), transduced NK cells were cultured for up to 21 days and analyzed for viability (A) and cell number (B) using trypan blue staining and a heamocytometer Neubauer chamber. Untransduced cells were cultured in parallel. Shown are results from two independent experiments starting with $4x10^4$ NK cells from two different donors which were transduced with two different vector stock batches of either huCAR19.CXCR4 (MOI of 2.94 and 3.09) or huCAR19 (MOI of 2.14 and 3.38). Mean value and standard deviation are shown. (C) The phenotype of gene modified $CD56^+$, $CAR19^+$ NK cells as well as untransduced $CD56^+$ cells were determined 6 days after isolation. Surface marker expression was analyzed by flow cytometry using a cocktail of antibodies including APC-Cy7 labeled anti-CD16 (clone 3G8), FITC conjugated anti-NKp46 (clone 9E2) and anti-CD57 (clone NK-1), PE-Vio770 labeled anti-NKp30 (clone AF29-4D12) and anti-CD62-L (clone 145/15) as well as PerCP labeled anti-NKG2C (clone #134591) and PerCP-Cy5 conjugated anti-NKG2D (clone 1D11). All antibodies are mouse anti-human. The bar diagram represents data from 3 independent experiments applying NK cells from 4 different donors. Mean value and standard deviation are shown (non-significant by unpaired t-test).

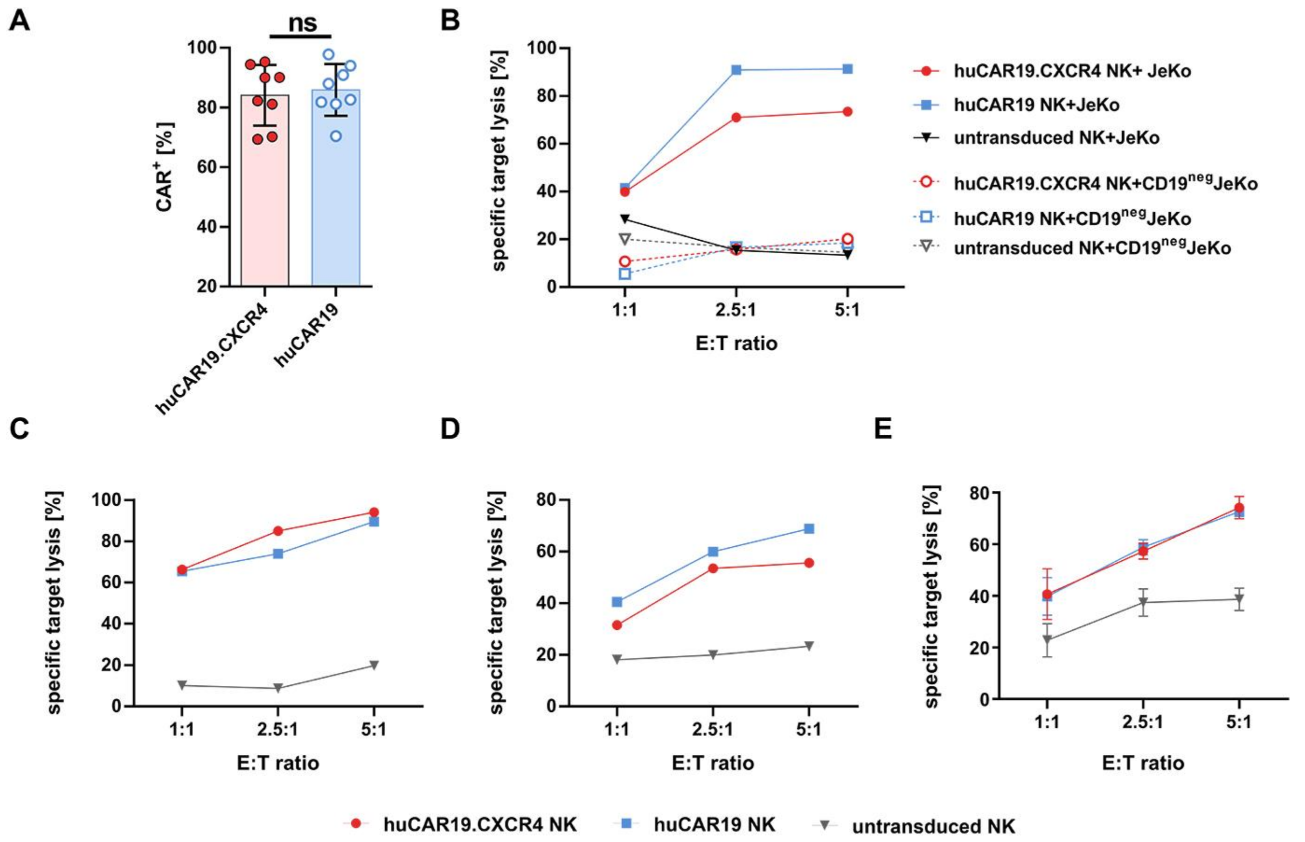

Supplementary Figure S4: Cytotoxic capacity of gene modified CAR NK cells against different tumor cell lines.

(A) Gene delivery efficiency of transduced NK cell batches applied for killing assay. Bar diagram shows mean value and SD as well as individual data points. (B-D) Specific cytotoxic function of genetically modified NK cells against CFSE-prelabled CD19 positive and negative tumor cell lines determined by flow cytometry based killing assay. Investigated were CD19-positive JeKo (B), Nalm-6 (C), SUP-B15 (D) and BV-173 (E) cells in three different effector to target ratios. JeKo cells, which have a genomic knockout for CD19 ($CD19^{neg}$JeKo), were used as non-target control in parallel to JeKo cells (B). The experiments were performed once with technical replicates as described for Nalm-6 in Figure 3A of the main manuscript.

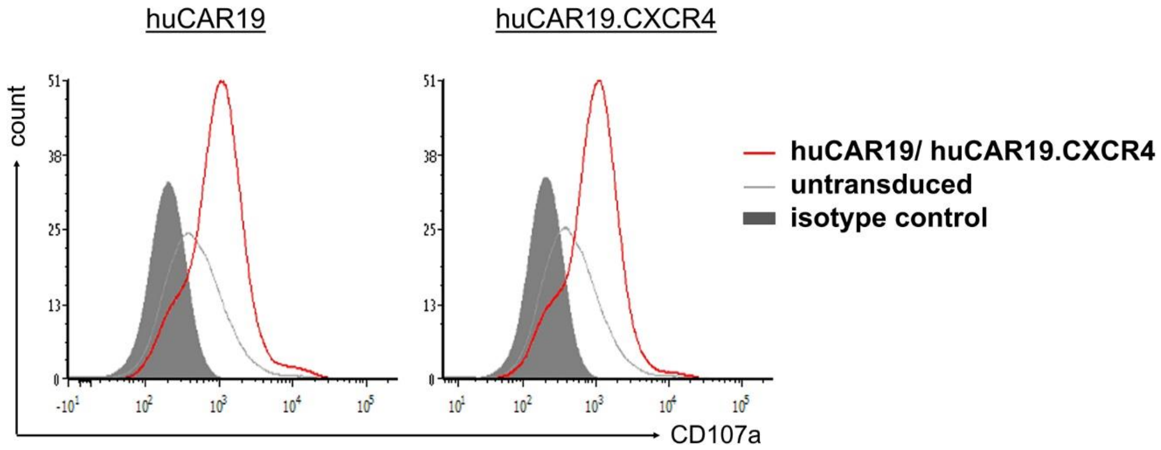

Supplementary Figure S5: **Histogram analysis of CD107a expression on NK cells.**

To determine NK degranulation, anti-CD107a antibody was added to co-culture of NK cells and CD19 positive Nalm-6 cells. After 4 hours of incubation at 37°C, detection of CD107a was performed by flow cytometry. Histograms are representative of one experiment shown in Fig. 4. Filled: isotype control, open grey: untransduced and open black: transduced.

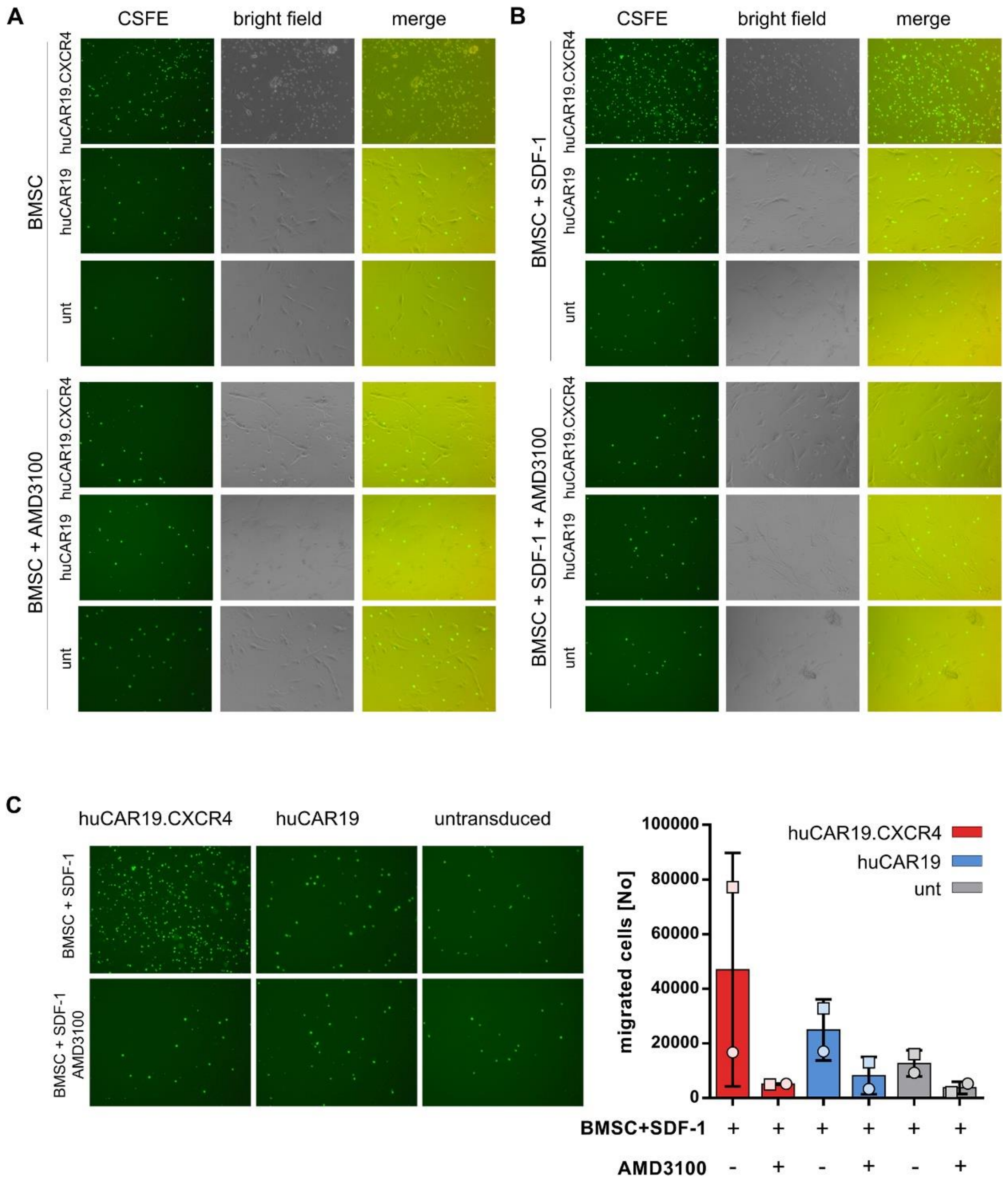

Supplementary Figure S6: Chemotaxis of huCAR.CXCR4 NK cells toward bone marrow stromal cells.

The migratory potential of genetically modified NK cells towards bone marrow stromal cells (BMSCs) in the absence (A) and presence (B/C) of 100 ng/ml of recombinant SDF-1 was evaluated in a migration assay using CSFE pre-labeled huCAR19.CXCR4, huCAR19, and untransduced NK cells with or without AMD3100 treatment. (A/B) Representative microscopic pictures of the lower wells after 2 hours of migration. Fluorescent (left), bright field (center) and merge (right) images are shown. (C) Quantitative data of migrated NK cells towards BMSC in the presence of recombinant SDF-1 determined by flow cytometry (left) and corresponding representative fluorescent images (right). Bar diagram shows individual results as well as mean values and standard deviation. The experiment was carried out once with purified NK cells from two different donors.

Supplemental Tables

Supplementary Table S1: Plasmid ratio for production of third generation lentiviral vectors. Plasmid amounts for transfection per 175 cm^2 culture area are shown [µg].

Plasmid	Ratio	Amount [µg]
**Transgene:**<br>- pEF1a.CAR19(4z)-P2A-CXCR4<br>- pEF1a.CAR19(4z)	5	17.5
**Envelope:**<br>VSVG (pMD2G)	1	3.5
**Helper :**<br>GAG/ POL (pMDlg)	3	10.5
Rev/tat (pRSV)	1	3.5

Supplementary Table S2: Vectors characteristics.

Vector	Stock No	Titer(t.u/ml)	Av size(nm)[(4]	PN/ml[(1)]	MOI[(2)]
EF1a.huCAR19-P2A-CXCR4-LV	1	$1.08x10^7$	117.9	$3.93x10^{11}$	0.54[(3)]
	2	$5.87x10^7$	112.8	$3.96x10^{11}$	2.94
	3	$6.18x10^7$	119.7	$6.25x10^{11}$	3.09
	4	$3.43x10^7$	132.4	$4.32x10^{11}$	1.72
**Mean± SD**		**$4.14x10^7$± ($2.38x10^7$)**	**120 ± 8.3**	**$4.64x10^{11}$ ± ($1.10x10^{11}$)**	**2.07±1.92**
EF1a.huCAR19-LV	1	$6.13x10^7$	109.2	$3.62x10^{11}$	3.07
	2	$4.27x10^7$	131.7	$1.78x10^{11}$	2.14
	3	$2.44x10^7$	122	$5.11x10^{11}$	1.22
	4	$6.75x10^7$	118.9	$4.25x10^{11}$	3.38
**Mean± SD**	**-**	**$4.90x10^7$ ± ($1.95x10^7$)**	**120.5 ± 9.3**	**$3.69x10^{11}$ ± ($1.4x10^{11}$)**	**2.4 ± 0.97**

(1) particle count per ml = PN/ml
(2) 2 µl vector stock per $4x10^4$ cells if not otherwise noted.
(3) This stock was used for transduction experiments including NK cell lines (Fig. 1C). 2 µl vector stock per $3x10^4$ cells were used.
(4) average particle size in nm = Av size (nm)
